# Hepatoprotective effect of peony total glucosides and the underlying mechanisms in diabetic rats

**DOI:** 10.1080/13880209.2017.1390589

**Published:** 2017-10-27

**Authors:** Ling-Ling Xia, Qi-Jin Zhu, Yong-Gui Wu

**Affiliations:** a Department of Infectious Diseases, The First Affiliated Hospital, Anhui Medical University, Hefei, P.R. China;; b Department of Nephrology, The First Affiliated Hospital, Anhui Medical University, Hefei, P.R. China

**Keywords:** Diabetes, liver, endoplasmic reticulum stress, inflammation

## Abstract

**Context:** Total glucosides of peony (TGP), compounds extracted from the dried roots of *Paeonia lactiflora* Pall, have been reported to have anti-inflammatory and antioxidative activities. However, the protective effect of TGP on liver injury and the underlying mechanisms remains unknown in diabetic rats.

**Objectives:** Current study investigates prevention of liver injury by TGP in diabetic rats and its mechanism was related to the inhibition of endoplasmic reticulum stress (ERS).

**Materials and methods:** Fifty adult male rats were randomly divided into: Normal group, diabetic group, TGP (50, 100 and 200 mg/kg/day) treatment groups (*n* = 10 per group). At the end of the 8th week, the liver was removed for biochemical and histological examinations.

**Results:** Compared with the diabetic group, administration of TGP at doses of 50, 100 and 200 mg/kg significantly prevented the increase of hepatic fibrosis score (ED_50_ 139.4 mg/kg). Compared with diabetic group, TGP at doses of 50, 100 and 200 mg/kg showed an inhibition on the increased macrophage infiltration. MCP-1 and TNF-α mRNA and protein expression were significantly increased in diabetic group compared with normal group; TGP administration caused significant reduction of high levels of MCP-1 and TNF-α mRNA as well as protein levels. Also, TGP at all doses showed an inhibition on the increased GRP78 levels, p-Perk levels and p-Eif2α levels in liver from diabetic group.

**Discussion and conclusions:** Our results indicate that TGP has potential as a treatment for diabetic liver injury attenuating liver lipid accumulation and inflammation as well as ERS induced by diabetic condition.

## Introduction

Diabetic mellitus is a serious risk for the development of renal, cardiovascular, retina and vascular disease, it is also related to liver injury. Diabetic liver injury is mainly associated with fatty change in the liver ranging from simple steatosis to steatohepatitis which may advance to cirrhosis and end-stage liver disease (Ohno et al. 2000; Farrell and Larter 2006; Welsh et al. 2012). The pathogenesis of diabetic liver injury is not completely understood, previous study has demonstrated that hepatic fat accumulation and oxidative stress play a critical role in the development of diabetic fatty liver (West 2000). Increasing evidence indicates that immune mediated inflammatory processes play a significant role in the pathophysiology of the diabetes milieu and its complications (Navarro-González and Mora-Fernández 2008). Experimental evidence has consistently demonstrated that macrophage infiltration was one of the most important events for the progression of diabetic liver injury (Wu et al. 2007). Endoplasmic reticulum stress (ERS) is a persistent state of pathophysiology that the endoplasmic reticulum unfolded protein response (UPR) or exceed the ERS ability. Previous study showed that the UPR was activated in several liver diseases, including obesity associated fatty liver disease, viral hepatitis and alcohol-induced liver injury, all of which were associated with steatosis (Hotamisligil 2010; Basseri and Austin 2012; Cnop et al. 2012; Fu et al. 2012; Ozcan and Tabas 2012), raising the possibility that ER stress-dependent alteration in lipid homeostasis was the mechanism that underlies the steatosis, recent studies show that ERS could induce inflammation (Zhang and Kaufman 2008; Watt et al. 2014).

**Figure 1.**
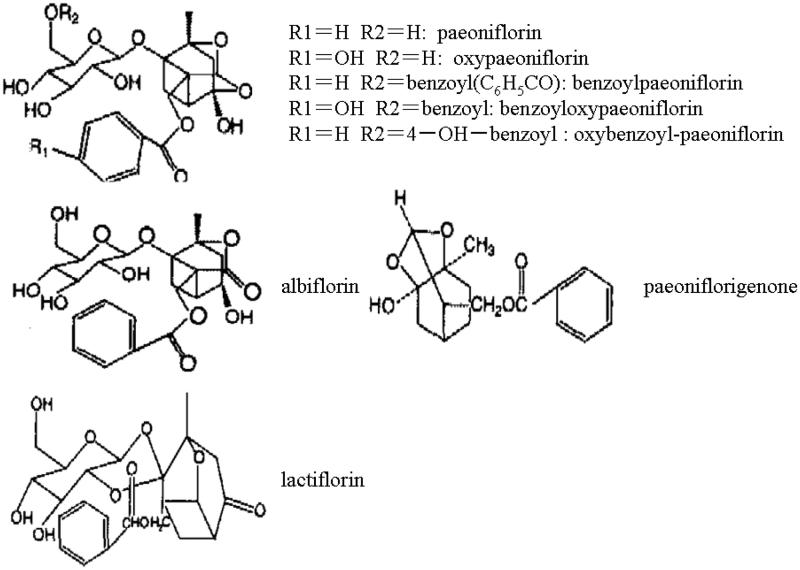
The chemical structures of major components in total glucosides of peony.

Traditional herbal medicines have been widely used for the treatment of diabetes and diabetic complications in Asian countries. It is suggested they have a promising future in diabetic prevention and treatment due to integrated effects. *Paeonia lactiflora* Pall root is one of the most important crude drugs in traditional Chinese medicine, which has been used for gynaecological problems and for cramp, pain and giddiness for over 1500 years in Chinese medicine. Total glucosides of peony (TGP) are active compounds extracted from the dried roots of *Paeonia lactiflora.* A reversed phase high performance liquid chromatography (HPLC) method was established for the simultaneous determination of eight major constituents of TGP, namely paeoniflorin, oxypaeoniflorin, benzoylpaeoniflorin, benzoyloxypaeoniflorin, oxybenzoyl-paeoniflorin, albiflorin, paeoniflorigenone, lactiflorin (Figure 1) (Xu et al. 1992). Among them, paeoniflorin was one of the main bioactive components of TGP. Studies demonstrate that the major metabolic pathway of paeoniflorin was intestinal bacteria transforming paeoniflorin into a major metabolite, paeonimetabolin I, which could be rapidly absorbed from the intestinal tract into the general circulation, then hydrolysis to the aglycone when given orally (He et al. 2002). The effect of TGP has been extensively proved for many years with an exact therapeutic effect on hepatitis, systemic lupus erythematosus, rheumatoid arthritis and hepatitis without evident toxic or side effects. The therapeutic effects include anti-inflammatory, antioxidative and immunoregulatory activities. TGP has also been approved by China Food and Drug Administration (CFDA) to enter market as a disease-modifying drug since 1998. Our previous study showed that TGP had obvious protection on the kidney of diabetic rats (Wu et al. 2009; Su et al. 2010; Zhou et al. 2012), but the effect of TGP on liver in diabetes had not been reported. As such, the present study was untaken to investigate whether and how TGP ameliorates early liver injury in streptozotocin (STZ)-induced diabetic rats.

## Materials and methods

### Drugs and reagents

**Figure 2.**
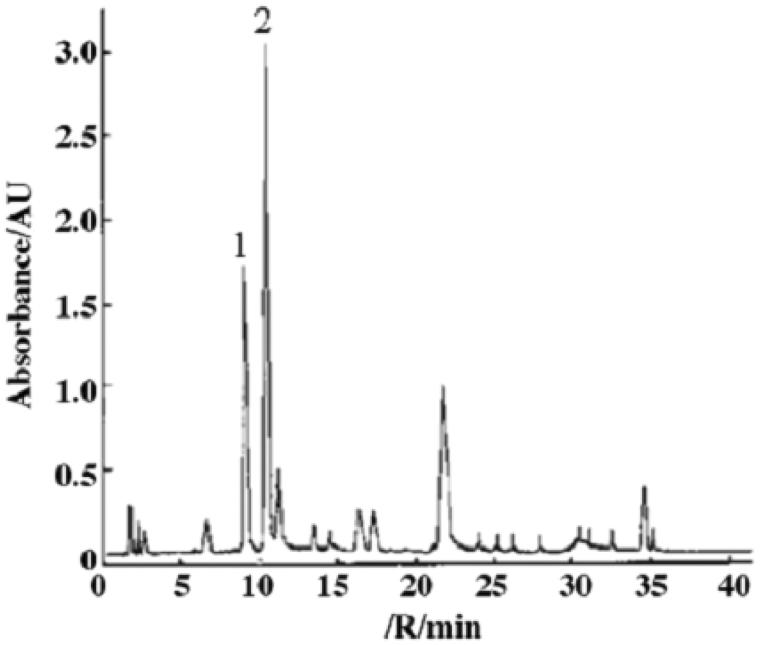
HPLC fingerprinting of total glucosides of peony. (1) Albiflorin; (2) paeoniflorin. Column: Supelcosil LC-18 (5 µm, 150 mm × 4.6 mm); solvent A: acetonitrile; solvent B: H20 (acidified to pH 3.0 with phosphoric acid); gradient: 10, 15, 18, 30, 35 and 40% of solvent A at 0, 5, 25, 27, 38, 40 and 50 min, respectively. Flow rate: 1.0 mL/min. Injection volume: 10 µL. Detection: 230 nm.

TGP was extracted and isolated from the root of *Paeonia lactiflora* by ethanol reflux, *n*-butanol extraction and macroreticular absorption resin chromatography. The extract was determined to contain 41.1% paeoniflorin by HPLC fingerprinting analysis (Figure 2). STZ was purchased from Sigma Chemical Co. (St. Louis, MO). Trizol reagent were purchased from Invitrogen (Carlsbad, CA), SYBR Green PCR master mix kit were purchased from Bio-Rad Laboratories (Hercules, CA). Revert Aid Premium First Strand cDNA Synthesis Kit were purchased from Promega (Madison, WI). Mouse anti-macrophage monoclonal (ED-1) antibody was from Abcam Biotechnology (Abcam, Cambridge, UK). Rabbit anti-MCP-1, TNF-α, GRP78, p-Perk and p-Eif2α antibodies were from Santa Cruz Biotechnology, Inc. (Santa Cruz, CA). Nitrocellulose membrane was obtained from Amersham Life Science (Little Chalfont, UK). The chemiluminescence kit was purchased from Amersham Life Science (Little Chalfont, UK). The immunohistochemistry kit (PV-9000) was purchased from Beijing Zhongshan Biotechnology Inc. (Zhongshan, China).

### Animals

Male Munich-Wistar rats (weight 180–200 g) were obtained from the Experimental Animal Center of Anhui Medical University (Hefei, China). Animals were housed in wire-bottomed cage under a 12 h light/dark cycle. Room temperature (about 24 ± 1 °C) and humidity (about 60%) were controlled automatically. They were allowed free access to standard laboratory chow and tap water. The Animal Ethics Committees of the Faculty of Anhui Medical University approved all experimental protocols, in accordance with ‘Principles of Laboratory Animal Care and Use in Research’ (Ministry of Health, Beijing, China).

### Experimental design

After several days of adaptation, rats were intraperitoneally injected with STZ diluted with citrate buffer (0.1 M, pH 4.0) at a dose of 65 mg/kg following overnight fasting. Two days later, the diabetic state was confirmed by measurement of tail blood glucose levels using a reflectance meter (One Touch II; Lifescan Ltd, Jinan, China). Blood glucose levels were measured twice a week. Diabetic rats were randomly divided into four groups (*n* = 10 per group), avoiding any inter-group differences in blood glucose levels. A normal group of rats (*n* = 10) was also included. The normal and control diabetic groups were administered 0.5% sodium carboxymethylcellulose (CMC-Na), while the other groups were administered TGP (suspended in 0.5% CMC-Na) orally at a dose of 50, 100 or 200 mg/kg daily using a stomach tube.

### Sample preparation

At eight weeks, rats were anesthetized with sodium pentobarbital (50 mg/kg IP), the right jugular artery was catheterized, and this arterial catheter was used for blood sampling. The liver sample was removed immediately and fixed in phosphate-buffered 10% formalin (pH 7.2) and processed in paraffin for subsequent histological assessment and immunohistochemical studies. The remaining liver was stored at −80 °C for evaluation of fat deposits, liver lipid parameter.

### Assay of blood glucose and lipid profile

Blood glucose was determined according to standard methods. The serum activity of aspartate aminotransferase (AST) and alanine aminotransferase (ALT) was estimated by commercially available kits (Nanjing Jiancheng Institute of Biotechnology, China). The total lipids were extracted from the liver tissue homogenate by the method of Folch et al. (1957). Triglycerides content in plasma and liver were estimated by the method of Foster and Dunn (1973); cholesterol was estimated by the method of Parekh and Jung (1970). Analysis of free fatty acid composition in lipid extract was performed in gas chromatography according to the method of Morrison and Simth (1964).

### Histological examination of liver

Formalin-fixed liver sections (4 µm) were stained with haematoxylin and eosin (HE) and Masson’s trichrome. In order to detect fat deposits in liver tissue, frozen sections from the liver were processed by Oil red O (Jingke Hongda Biological Technique Ltd., Beijing, China) staining. Morphological analysis was assessed by computerized image analysis system (Beijing Aeronautic and Aerospace University, China) on 10 microscopic fields per section examined at a magnification of 100×, with the observer blind to the study group. Histological lesions (fatty changes and fibrosis) were evaluated according to the following scale: 0: absent; 1: mild (involving 10% per microscopic field); 2: moderate (>11 and =25%); 3: severe (>26 and =50%) or 4: very severe (>50%) (Toblli et al. 2002).

### Immunohistochemistry

Immunoperoxidase staining for ED-1 (macrophage marker) was performed on 10% formalin-fixed paraffin sections (2 μm), 3% hydrogen peroxide closing endogenous peroxidase, antigen retrieval [microwave oven heating in 0.1 M sodium citrate (pH 6.0 for 10 min)]. Tissue sections were incubated with 10% normal goat serum for 10 min followed by an overnight incubation with anti-ED-1 antibody (1:100) in 10% normal goat serum at 4 °C. The sections were washed, incubated with horseradish peroxidase (HRP)-labelled goat anti-mouse antibody for 20 min at 37 °C, and developed with 3,3-diaminobenzidine (DAB, Sigma, St. Louis, MO) to produce a brown colour. Sections were counterstained with haematoxylin. Immunohistochemistry for ED-1 was assessed on 10 consecutive microscopic fields per section examined at a magnification of 100×. Amount of ED-1 was graded according to the following scale: 0: absent; 1: mild (involving 10% per microscopic field); 2: moderate (>11 and =25%); 3: severe (>26 and =50%) or 4: very severe (>50%) (de Cavanagh et al. 2001). All scoring was performed on blinded slides.

### qRT-PCR

Total RNA was extracted from the liver using Trizol reagent according to the manufacturer's instructions. RNA (1 µg) was reverse-transcribed to cDNA using A3500 Reverse Transcription System. The cDNA was amplified by real-time PCR with iTaq™ Universal SYBR^®^ Green Supermix, and the housekeeping gene GAPDH was used as the internal control. SYBR Green assays were performed in triplicate on a CFX Connect™ real-time instrument. The primers to detect mRNA were: GAPDH: forward primer 5-GGTGAAGGTCGGTGTGAACG-3, reverse primer 5-CTCGCTCCTGGAAGATGGTG-3; TNF-α: forward primer 5-GCTGAGCTCAAACCCTGGTA-3, reverse primer 5-CGGACTCCGCAAAGTCTAAG-3; MCP-1: forward primer 5-TTGACCCGTAAATCTGAAGCTAAT-3, reverse primer 5-TCACAGTCCGAGTCACACTAGTTCAC-3. Relative expression was calculated by using the 2^−ΔΔCt^ method with values normalized to the reference gene GAPDH.

### Western blot analysis

Liver samples were homogenized in RIPA buffer (PBS, 1% NP-40, 0.5% sodium deoxycholate, 0.1% SDS, 100 µg/mL aprotinin, 100 µg/mL phenylmethylsulfonyl fluoride, sodium orthovanadate) at 4 °C throughout all procedures. The protein concentration was measured by the dye binding assay of Bradford and separated by 15% SDS-PAGE. Separated proteins were transferred to nitrocellulose membranes and the blots were blocked using 5% skim milk in PBST. The membranes were hybridized with polyclonal rabbit anti-rat MCP-1 (diluted 1:1000), TNF-α (diluted 1:1000), GRP78 (diluted 1:1000), p-Perk (diluted 1:1000) and p-Eif2α (diluted 1:1000), and then incubated with a HRP-labelled goat anti-rabbit IgG (diluted 1:500). Immunoreactive bands were visualized using the ECL detection system (Amersham, UK). Housekeeping protein β-actin was used as a loading control. Positive immunoreactive bands were quantified densitometrically (Leica Q500IW image analysis system, Wetzlar, Germany) and expressed as an intensity ratio.

### Statistics

Data were expressed as the mean ± SEM. One-way analysis of variance (ANOVA) with pairwise comparisons according to the Tukey method was used in this study. Differences were considered significant if the *p* value was less than 0.05.

## Results

### Clinical and metabolic parameters

As shown in Table 1, body weight was significantly decreased in diabetic rats, TGP could not prevented the decline in body weight. The liver weight of diabetic rats increased significantly, while oral administration of TGP (50, 100 and 200 mg/kg) for 8 weeks attenuated the abnormal increased liver weight. Compared with normal group, diabetic rats developed elevated plasma triglycerides and cholesterol levels, administration of TGP could not change the levels of cholesterol and triglycerides. The activities of AST and ALT were increased significantly in the diabetic group of rats when compared to control group of rats. Oral treatment of TGP normalized the activities of these enzymes to near normal when compared to control group of rats (Table 2).

**Table 1.** Effects of TGP on triglycerides, and total cholesterol and free fatty acids in liver of diabetic rats induced by STZ.

Group	Dose (mg/kg)	Blood glucose(mg/dl)	Body weight (g)	Liver weight(g/100 g BW)	Plasma triglycerides(mg/dl)	Plasma cholesterol(mg/dl)
Normal	−	123.53 ± 29.19	458 ± 27.47	4.25 ± 0.36	102.66 ± 13.28	42.96 ± 3.09
Control diabetic	−	469.07 ± 74.58**	271.75 ± 16.86**	6.88 ± 0.58**	150.45 ± 14.16*	47.99 ± 7.35*
Diabetic + TGP	50	445.04 ± 77.43	281.75 ± 25.01	5.12 ± 0.42#	148.68 ± 11.51	47.21 ± 4.64
	100	470.49 ± 75.47	266.4 ± 27.87	4.68 ± 0.36#	145.14 ± 12.39	46.05 ± 5.03
	200	484.16 ± 75.65	318.0 ± 17.8	4.56 ± 0.40#	160.19 ± 15.93	46.44 ± 5.02

Values are expressed as means ± SEM. Number of rats in each group was 10.

* *p*< 0.05.

** *p*< 0.01 indicates a significant difference from normal.

# *p*< 0.05 indicates a significant difference from control diabetic.

**Table 2.** Effects of TGP on serum ALT and AST levels of diabetic rats induced by STZ.

Group	Dose (mg.kg^−1^)	AST (U/L)	ALT (U/L)
Normal	−	100.30 ± 5.36	28.53 ± 3.24
Control diabetic	−	130.36 ± 10.76**	55.47 ± 5.18**
Diabetic + TGP	50	120.20 ± 6.28#	50.74 ± 7.48#
	100	115.58 ± 8.32#	45.79 ± 5.35#
	200	103.49 ± 7.16##	30.64 ± 2.13##

Values are expressed as means ± SEM. Number of rats in each group was 10.

** *p*< 0.01 indicates a significant difference from normal.

# *p*< 0.05.

## *p*< 0.01 indicates a significant difference from control diabetic.

### Lipid content in the liver

Table 3 represents the lipid content in liver of normal and diabetic rats. STZ-induced diabetes resulted in increased levels of triglycerides, cholesterol and free fatty acids. Administration of TGP (50, 100 and 200 mg/kg) to diabetic rats resulted in a significant decrease in the levels of liver lipid contents when compared to STZ-diabetic rats.

**Table 3.** Effects of TGP on triglycerides, and total cholesterol and free fatty acids in liver of diabetic rats induced by STZ.

Group	Dose (mg kg^−1^)	Triglycerides (mg/g)	Cholesterol (mg/g)	Free fatty acids (mg/g)
Normal	−	7.10 ± 0.46	7.45 ± 0.42	1.32 ± 0.16
Control diabetic	−	11.36 ± 0.76**	9.48 ± 0.88*	2.76 ± 0.34*
Diabetic + TGP	50	8.20 ± 0.58#	8.32 ± 0.68#	1.86 ± 0.15#
	100	7.78 ± 0.52#	7.79 ± 0.65#	1.74 ± 0.16#
	200	7.49 ± 0.56#	7.64 ± 0.73#	1.65 ± 0.18#

Values are expressed as means ± SEM. Number of rats in each group was 10.

* *p*< 0.05.

** *p*< 0.01 indicates a significant difference from normal.

# *p*< 0.05 indicates a significant difference from control diabetic.

### Histological examination in the liver

**Figure 3.**
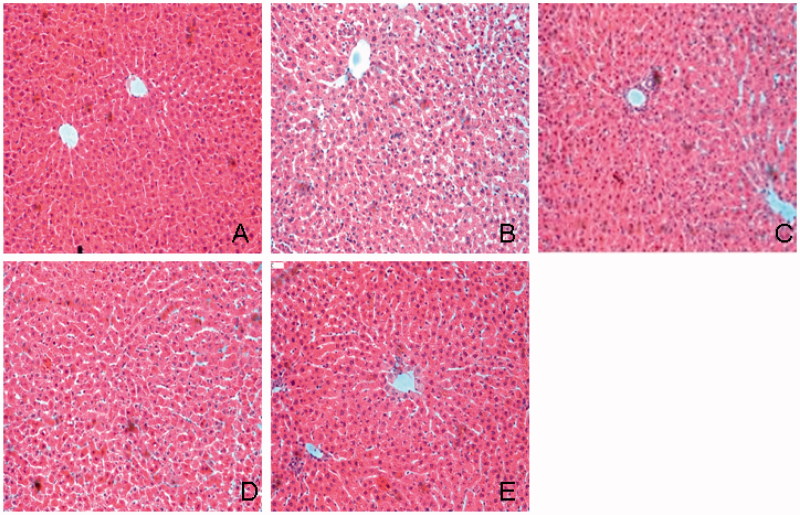
Histological examination of liver in HE staining. (A) Normal; (B) control diabetic; (C) diabetic + TGP 50 mg/kg; (D) diabetic + TGP 100 mg/kg; (E) diabetic + TGP 200 mg/kg. Original magnification 100×.

**Figure 4.**
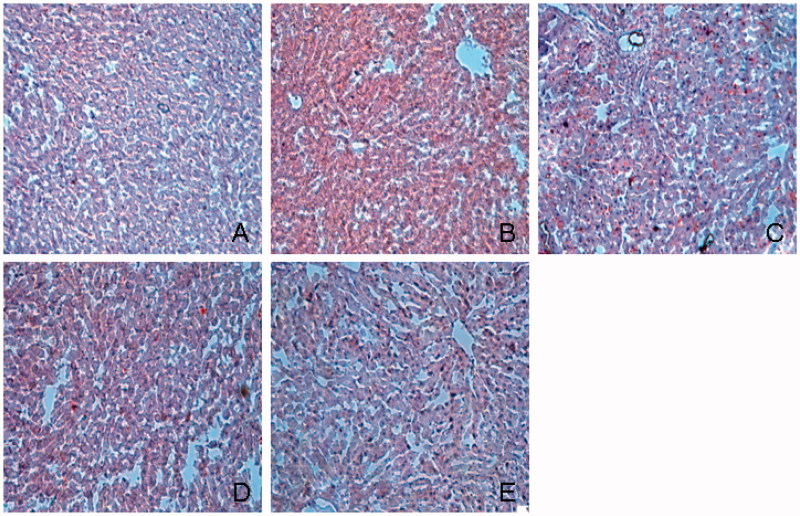
Histological examination of liver in Oil red O staining. (A) Normal; (B) control diabetic; (C) diabetic + TGP 50 mg/kg; (D) diabetic + TGP 100 mg/kg; (E) diabetic + TGP 200 mg/kg. Original magnification 100×.

**Figure 5.**
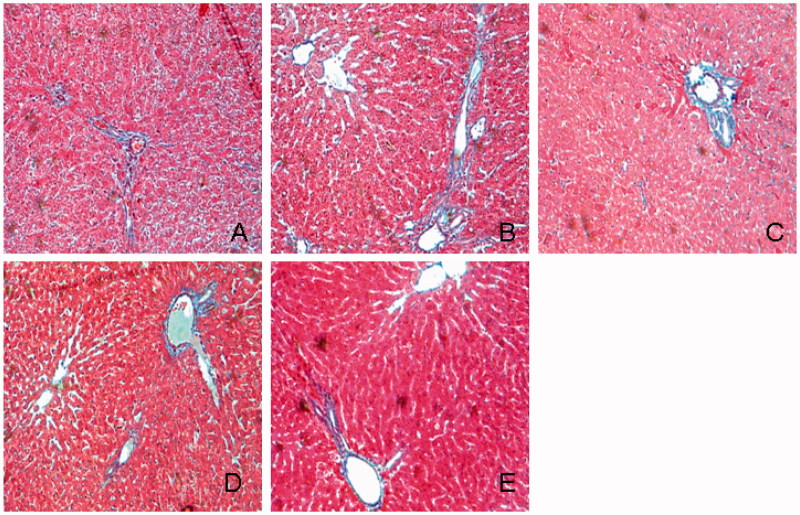
Histological examination of liver in Masson’s trichrome staining. (A) Normal; (B) control diabetic; (C) diabetic + TGP 50 mg/kg; (D) diabetic + TGP 100 mg/kg; (E) diabetic + TGP 200 mg/kg. Original magnification 100×.

HE staining showed that liver cell fatty degeneration was increased in diabetic group and significantly reduced in TGP-treated rats (Figure 3). Figure 4 shows histological observation of liver sections stained with Oil red O. Oil red O staining score in diabetic group was significantly higher than that in normal group (2.67 ± 0.86 vs. 0.42 ± 0.16, *p* < 0.01), TGP 50, 100, 200 mg/kg treatment group scores were 1.13 ± 0.78, 1.08 ± 0.67 and 0.88 ± 0.64, respectively, which were significantly (*p* < 0.05) lower than those in diabetic group. Figure 5 shows histological observation of liver sections stained with Masson’s trichrome. Masson staining showed hepatic fibrosis score was higher than that in normal group (1.86 ± 0.64 vs. 0.46 ± 0.34, *p*<  0.01), hepatic fibrosis scores in TGP 50, 100 and 200 mg/kg treatment group were 1.28 ± 0.52, 1.16 ± 0.37 and 0.86 ± 0.56, respectively, which were significantly (*p* < 0.05) lower than those in diabetic group, particularly at the dose of 200 mg/kg. ED_50_ value was 139.4 mg/kg.

### Macrophage infiltration in the liver

**Figure 6.**
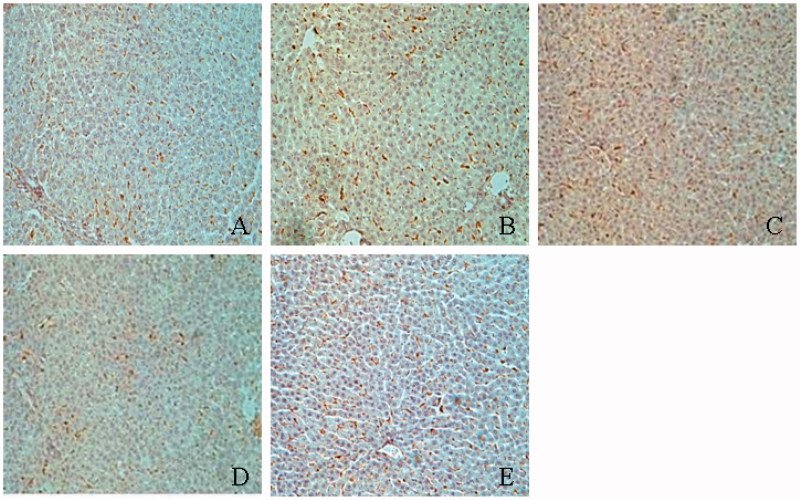
Immunostaining of ED-1 positive cell in the liver. (A) Normal; (B) control diabetic; (C) diabetic + TGP 50 mg/kg; (D) diabetic + TGP 100 mg/kg; (E) diabetic + TGP 200 mg/kg. Original magnification 100×.

A small number of macrophage (ED-1-positive cell) were observed in liver from normal rats, liver from diabetic rats presented increased macrophage infiltration, especially in the perivascular and pericanalicular areas of the liver (Figure 6). Macrophage score in diabetic rats was higher than that in the normal rats (2.68 ± 0.88 vs. 0.64 ± 0.42, *p* < 0.01) and these changes were largely blocked by TGP with 50, 100 and 200 mg/kg (1.58 ± 0.42, 1.32 ± 0.36 and 0.78 ± 0.32 vs. 2.68 ± 0.88, *p* < 0.05, 0.01).

### MCP-1 and TNF-α mRNA and protein expression in the liver

**Figure 7.**
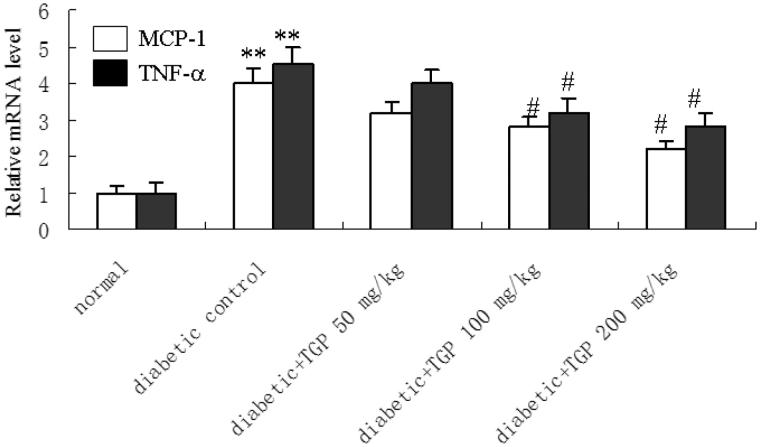
MCP-1 and TNF-α mRNA expression in the liver. Quantitative real time PCR analyse MCP-1 and TNF-α mRNA expression in the liver. **p < 0.01 vs. normal; #*p* < 0.05, vs. control diabetic.

**Figure 8.**
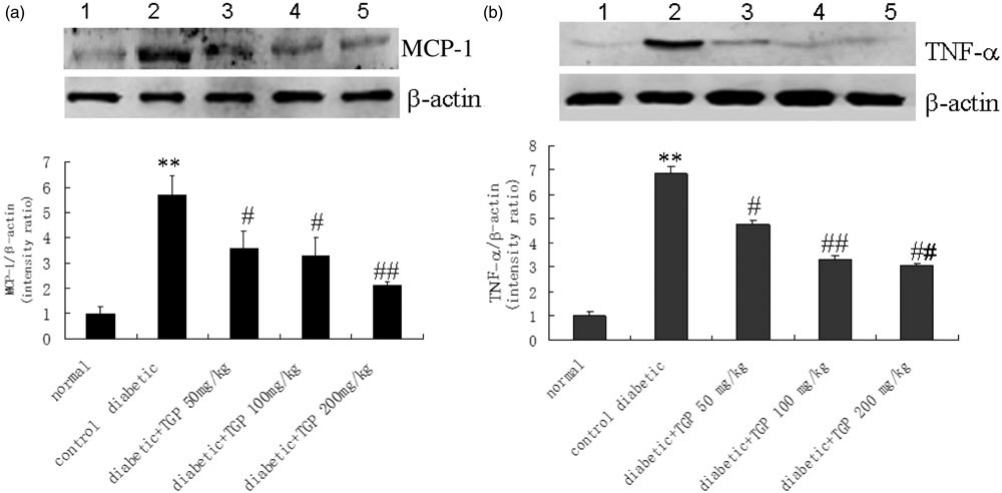
(a) Western blot analysis of MCP-1 in liver issue in five groups of rats. (1) Normal; (2) control diabetic; (3) diabetic + TGP 50 mg/kg; (4) diabetic + TGP 100 mg/kg; (5) diabetic + TGP 200 mg/kg. **p < 0.01 vs. normal; #*p* < 0.05, ##*p* < 0.01 vs. control diabetic. (b) Western blot analysis of TNF-α in liver issue in five groups of rats. (1) Normal; (2) control diabetic; (3) diabetic + TGP 50 mg/kg; (4) diabetic + TGP 100 mg/kg; (5) diabetic + TGP 200 mg/kg. **p < 0.01 vs. normal; #*p* < 0.05, ##*p* < 0.01 vs. control diabetic.

Figure 7 shows the effect of TGP on MCP-1 and TNF-α mRNA expression in the liver from diabetic rats induced by STZ. Compared with normal group, MCP-1 and TNF-α mRNA expression were significantly increased in control diabetic group, TGP administration with 50, 100 and 200 mg/kg resulted in a concentration-dependent decrease in the MCP-1 and TNF-α mRNA levels. Densitometric analysis of the Western blot showed MCP-1 and TNF-α protein expression were significantly increased in control diabetic group, but they were significantly reduced by TGP administration with 50, 100 and 200 mg/kg (Figure 8(a,b)).

### GRP78 expression in the liver

**Figure 9.**
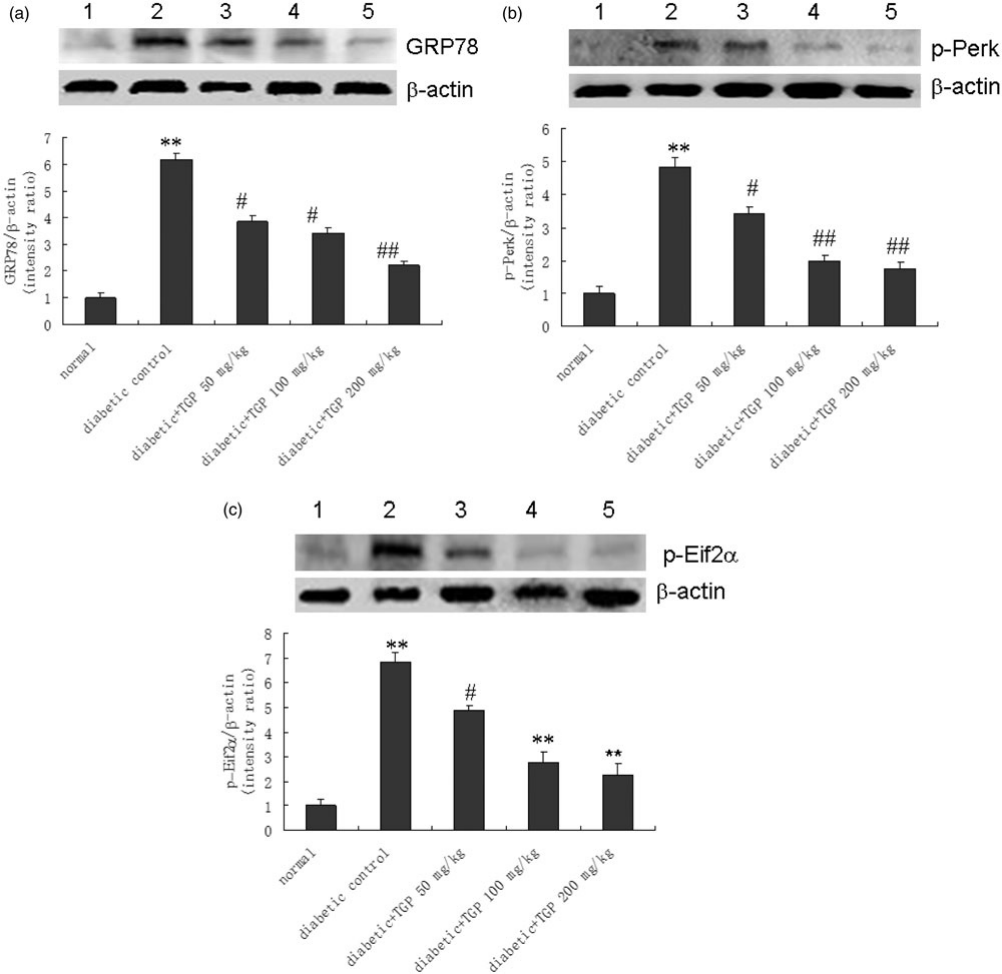
(a) Western blot analysis of GRP78 in liver issue in five groups of rats. (1) Normal; (2) control diabetic; (3) diabetic + TGP 50 mg/kg; (4) diabetic + TGP 100 mg/kg; (5) diabetic + TGP 200 mg/kg. **p < 0.01 vs. normal; #*p* < 0.05, ##*p* < 0.01 vs. control diabetic. (b) Western blot analysis of p-Perk in liver issue in five groups of rats. (1) Normal; (2) control diabetic; (3) diabetic + TGP 50 mg/kg; (4) diabetic + TGP 100 mg/kg; (5) diabetic + TGP 200 mg/kg. **p < 0.01 vs. normal; #*p* < 0.05, ##*p* < 0.01 vs. control diabetic. (c) Western blot analysis of p-Eif2α in liver issue in five groups of rats. (1) Normal; (2) control diabetic; (3) diabetic + TGP 50 mg/kg; (4) diabetic + TGP 100 mg/kg; (5) diabetic + TGP 200 mg/kg. **p < 0.01 vs. normal; #*p* < 0.05, ##*p* < 0.01 vs. control diabetic.

Figure 9(a) shows the effect of TGP on GRP78 expression in the liver from diabetic rats induced by STZ. Densitometric analysis of the Western blot showed a 5.2-fold higher in the amount of GRP78 from control diabetic rats with respect to normal rats, but it was significantly reduced by TGP administration with 50, 100 and 200 mg/kg by 38.1, 44.4 and 64.1%, respectively.

### p-Perk expression in the liver

p-Perk expression in the liver is shown in Figure 9(b). Protein band intensities of p-Perk expression were corrected by β-actin. Control diabetic rats had 3.8-fold higher expression of p-Perk than normal rats; TGP at 50, 100 and 200 mg/kg reduced p-Perk protein expression by approximately 29.2, 59.0 and 63.4%, respectively.

### p-Eif2α expression in the liver

Liver p-Eif2α expression is shown in Figure 9(c). Western blot analysis showed that the amount of immunoreactive peptide was increased in the liver of control diabetic rats compared to that from normal rats. Densitometric analysis of the Western blot showed a 5.8-fold increase in the amount of p-Eif2α from control diabetic rats compared to that in normal rats; treatment with TGP at 50, 100 and 200 mg/kg reduced p-Eif2α protein expression by 28.8, 59.6 and 67.1%, respectively.

## Discussion

The pathogenesis of diabetic liver injury has not been fully elucidated, because of insufficient insulin secretion and insulin resistance during diabetes, patients happened to hyperglycaemia, hyperlipidaemia, hyperaminoacidemia based on the disturbance of carbohydrate metabolism. Hyperlipidaemia in diabetic patients is mainly hypertriglyceridemia which can lead to liver fatty infiltration. However, diabetes liver fatty infiltration is only a starting factor in liver injury; oxidative stress induced by fatty infiltration is the fundamental factors in diabetic liver injury (Martindale and Holbrook 2002; Maritim et al. 2003; Newsholme et al. 2007). Oxidative stress might cause lipid peroxidation with increased mitochondrial uncoupling protein expression which could induce cytokine, growth factor and ligands activation, they further cause inflammation and fibrosis (Evans et al. 2002). Our previous study showed that type 1 diabetes might occur hepatic steatosis and fibrosis in the early stage, at the same time, the level of malondialdehyde (MDA) was significantly increased in tissue of diabetic liver, antioxidant superoxide dismutase (SOD), catalase (CAT), reduced glutathione (GSH), glutathione peroxidase (GSH-PX) were obviously decreased (Wu et al. 2007). Recently, Martinović et al. (2012) reported that DNA damage in liver was significantly increased accompanied by elevated oxidative stress and decreased antioxidant ability in STZ-induced diabetic rats, and with the increasing DNA damage in liver, inflammatory mediators TNF-α significantly was elevated, which also significantly increased activation of NF-κB, JAK-STAT and JNK signalling pathways.

In the present study, 8 weeks after single STZ injection, rats exhibited hyperglycaemia and increased lipid profile in plasma and liver, histological observation showed lipid accumulation and scant fibrosis in liver. Our study demonstrated that early diabetic state was related to liver fatty change and lesion, this result was consistent with the findings of de Cavanagh et al. (2001). Although hyperglycaemia and hyperlipidaemia were known risk factor for fatty infiltration of the liver, only treatment of hyperglycaemia might be helpful in the management of diabetic liver injury, use of lipid-lowering agents for hyperlipidaemia was contradictory, many lipid-lowering agents could promote lipid to liver for metabolism, and accelerated fatty infiltration of the liver and hasten liver damage (Busaranogla et al. 1999). Therefore, there was vital significance to further explore interventions on diabetic liver injury.

In recent years, research has shown that TGP display liver protective effect on a variety of liver injury including chemical, alcoholic, and nonalcoholic, immunity, drug-induced, biological model (Liu et al. 2006; Qin and Tian 2011; Chen et al. 2013), but TGP has not been reported for protection on diabetic liver injury. Our present study shows that TGP can reduce cholesterol, triglyceride and free fatty acid levels in the tissue of diabetic liver and improve the pathological damage and liver fatty infiltration and decrease liver tissue macrophage infiltration. Hyperglycaemia is associated with liver dysfunction in IDDM. Elevated activities of serum ALT and AST are a common sign of liver diseases. In the present study, our findings of elevated serum ALT and AST levels are in agreement with the findings of Liu et al. (2013). The increase in ALT and AST activities may be due to the cellular damage in the liver caused by STZ induced diabetes. However, TGP-treated diabetic rats, significantly lower the levels of these hepatic enzymes suggesting that TGP may protect the hepatic tissue damage caused by STZ-induced diabetes. But, TGP did not show any significant effect on body weight, blood glucose, plasma triglyceride or plasma cholesterol, mechanism might be further studied. In addition, our present study showed that the liver from the diabetic group presented macrophage infiltration, especially in the perivascular and pericanalicular areas, and TGP treatment could inhibit the recruitment of macrophages.

Liver cells have the active function of metabolism, with a number of rich endoplasmic reticulum, dysfunction of the endoplasmic reticulum is bound to the liver function and lead to illness. In the pathogenesis of nonalcoholic fatty liver, large amounts of free fatty acids accumulated in the liver cells and induced lipid peroxidation, which could bring about liver cells apoptosis and necrosis, in this process, strong lipid peroxidation is a predisposing factor of ERS (Hotamisligil 2010; Fu et al. 2012). Compared with the control group, Wistar rats fed by the high saturated fatty acid occur fatty degeneration of liver, during the process, ALT and AST level increased, ERS protein CHOP, GRP78, XBP-1 expression was also increased and Caspase 3 protein activity related to apoptosis is elevated (Zhao et al. 2013). Using a multilayered genetic approach, Rutkowski et al. (2008) found that mice with genetic ablations of either ER stress sensing pathways (ATF6α, eIF2α, IRE1α), or of ER quality control (p58IPK), shared a common dysregulated response to ER stress that included the development of microvesicular steatosis. The rescue of ER protein processing capacity by the combined action of UPR pathways during stress prevented the suppression of a subset of metabolic transcription factors that regulated lipid homeostasis. This suppression occurs in part by unresolved ER stress perpetuating expression of the transcriptional repressor CHOP. As a consequence, metabolic gene expression networks were directly responsive to ER homeostasis. Their results reveal an unanticipated direct link between ER homeostasis and the transcriptional regulation of metabolism and suggested mechanisms by which ER stress might underlie microvesicular steatosis. High fat diet fed rats changed into semi feed food restricted feeding could effectively reduce the expression of GRP78 in rat liver; fracture of the endoplasmic reticulum swelling was restored (Wei et al. 2006).

In recent years, studies show that ERS is the main factor to cause the metabolic inflammation such as obesity, insulin resistance, type 2 diabetes, in the process of ERS, three UPR pathways are activated. There is a connection between UPR and inflammatory signal transduction pathway through a variety of mechanisms including NF-κB and mitogen activated protein kinase JNK, ROS and endoplasmic reticulum calcium release, etc. Our study shows that ERS related protein GRP78 and PERK protein expression significantly increased in diabetic liver tissue.

At the same time, the inflammatory infiltration of macrophages was significantly increased and pro-inflammatory cytokine such as MCP-1 and TNF-α mRNA and protein expression were also markedly elevated in diabetic liver. TGP administration with 50, 100 and 200 mg/kg resulted in significant decrease in liver macrophage infiltration as well as MCP-1 and TNF-α mRNA and protein levels. Our study suggests ERS may cause fat liver and inflammatory mechanisms involved in diabetic liver damage, TGP could obviously decrease GRP78, p-Perk and p-Eif2α protein expression in diabetic liver tissue, which suggests that TGP has liver protection through the inhibition of ERS and inflammation, but the detailed mechanism is still needed, further study confirmed *in vitro* study.

Taking together, our study demonstrated that lipid accumulation in the liver was increased in early diabetic state, they were accompanied with ERS and inflammation. TGP has potential as a treatment for diabetic liver injury through attenuating liver ERS and inflammation.
